# Liver metastases and the efficacy of immune checkpoint inhibitors in advanced lung cancer: A systematic review and meta-analysis

**DOI:** 10.3389/fonc.2022.978069

**Published:** 2022-10-18

**Authors:** Handai Xia, Wengang Zhang, Yuqing Zhang, Xiaoling Shang, Yanguo Liu, Xiuwen Wang

**Affiliations:** Department of Medical Oncology, Qilu Hospital of Shandong University, Jinan, China

**Keywords:** immune checkpoint inhibitors, liver metastases, lung cancer, meta-analysis, efficacy

## Abstract

**Background:**

Liver metastasis is the most common type of lung cancer metastasis, and is a significant prognostic factor in lung cancer. However, the effect of liver metastases on the efficacy of immune checkpoint inhibitors (ICIs) remains inconsistent and controversial. The aim of this study was to explore the relationship between liver metastases and ICI efficacy in patients with advanced lung cancer based on data from randomized controlled trials (RCTs) and observational studies.

**Methods:**

PubMed, EMBASE, Cochrane Library databases, conference proceedings, as well as grey literature websites were searched for eligible studies without language restrict ion. Study quality was assessed using Cochrane tools and the Newcastle–Ottawa Quality Assessment Scale (NOS). Outcomes of interest were overall survival (OS) and progression-free survival (PFS). The difference in efficacy between patients with and without liver metastases was calculated by pooling ratios of hazard ratios (HR), as calculated using the deft approach.

**Results:**

A total of 16 RCTs and 14 observational trials were included. Analyses of RCTs revealed a survival benefit for ICI treatment (i.e., ICI monotherapy, ICI + Chemotherapy, dual ICI therapy and dual ICI + Chemotherapy) versus standard therapies among non-small cell lung cancer (NSCLC) patients with liver metastases (PFS HR, 0.77; 95%CI, 0.61–0.97; OS HR, 0.78; 95%CI, 0.68–0.90). NSCLC patients with liver metastases achieved less PFS benefit and comparable OS benefit from ICI treatment compared with those without liver metastases (ratios of PFS–HRs, 1.19; 95%CI, 1.02–1.39; P=0.029; Ratios of OS–HRs, 1.10; 95%CI, 0.94–1.29; P=0.24). For patients with small cell lung cancer (SCLC), ICI treatment achieved a marginal effect on patients with liver metastases as compared with standard therapies (OS HR, 0.94; 95%CI, 0.73–1.23). SCLC patients with liver metastases benefited less from ICI treatment than patients without liver metastases (ratio of OS–HRs, 1.22; 95%CI, 1.01–1.46; P=0.036). In real-world data analysis, liver metastasis could be used as an independent prognostic risk factor, increasing the risk of death by 21% in lung cancer patients receiving ICI treatment compared with those without liver metastases (OS HR, 1.21; 95%CI, 1.17–1.27; P<0.0001). Subgroup analysis confirmed that this association was not modified by race (Asian vs. Western) or number of treatment lines.

**Conclusions:**

The presence of liver metastases does not significantly influence the OS benefit of ICIs in patients with NSCLC. However, a small amount of data shows that liver metastasis restrains the magnitude of OS benefit in patients with SCLC. Liver metastasis has potential as an independent prognostic risk factor for lung cancer patients receiving ICI treatment in clinical practice.

**Systematic Review Registration:**

https://www.crd.york.ac.uk/PROSPERO/, identifier (CRD42022306449).

## Introduction

Lung cancer is the most common malignant tumor and is responsible for the largest number of cancer-related deaths worldwide (1). Metastasis is the main cause of death in patients with lung cancer and around 40% of patients have metastatic disease at the time of diagnosis (2). The liver is one of the most common metastatic site of advanced lung cancer due to its abundant blood supply, leading to poor therapeutic effect and prognosis (3). Among lung cancer patients with isolated organ metastasis, patients with liver metastases have the worst prognosis, with about 6 months of cancer-caused survival (4).

Treatment options for patients with liver metastases remain limited (3). Chemotherapy has traditionally been the standard treatment for lung cancer patients with liver metastases, but the majority of those patients did not respond well to chemotherapy. For example, a retrospective analysis published in 2000 reviewed a cohort of 261 patients with non-small cell lung cancer (NSCLC) receiving first-line chemotherapy, and found that patients with liver metastases had 2.28-times the risk of death compared with patients without liver metastases (P=0.0014) (5). In addition, some patients are unable to complete the recommended cycles of chemotherapy due to liver dysfunction (6).

Recently, immune checkpoint inhibitors (ICIs) targeting the programmed cell death protein 1/ligand 1 (PD-1/PD-L1) axis have changed the treatment landscape for patients with a multitude of advanced cancers, including lung cancer (7–10). Anti‐PD‐1/PD‐L1 antibodies, including pembrolizumab, atezolizumab and durvalumab, block PD-1/PD-L1 pathway and enhance T-cell response as well as immune system recognition. More recently, García-Mulero and colleagues (11) analyzed 374 metastatic samples and found that liver metastases from various primary tumors, including lung cancer, have a lower infiltration of cytotoxic T lymphocytes (CTLs) compared with metastases in the lung, bone and brain. Some single-center reports have also identified liver metastasis as an independent prognostic factor of poor clinical outcomes of lung cancer patients who received PD-1/PD-L1 inhibitors (12, 13). However, the 2021 final analysis of KEYNOTE-189 indicated that patients with liver metastases could benefit from first-line pembrolizumab plus chemotherapy treatment, although the benefit was lower than in patients without liver metastases (14). Similarly, a pooled analysis of CheckMate 017 and CheckMate 057 with more than 3 years’ follow-up demonstrated the superiority of nivolumab over docetaxel in NSCLC patients with liver metastases (15). Taken together, the survival benefit of ICIs in lung cancer patients with liver metastases remains equivocal.

The relationship between liver metastases and ICI efficacy in advanced lung cancer patients is not fully understood. To address this gap in knowledge, we performed a meta-analysis of recently completed trials and clinical data, to comprehensively assess the influence of liver metastases on the efficacy of ICIs in advanced lung cancer.

## Materials and methods

### Search strategy

We conducted a systematic literature search with a predetermined protocol according to the Preferred Reporting Items for Systematic Reviews and Meta-Analyses (PRISMA) guidelines (16). The PubMed, EMBASE and Cochrane Library were systematically reviewed for randomized controlled trials (RCTs) or observational trials investigating the immunotherapy (i.e., nivolumab, pembrolizumab, atezolizumab, durvalumab, avelumab, sintilimab, tislelizumab) in advanced lung cancer published between January 1,2000 and March 10, 2022 with no language restrictions. We also reviewed the reference lists of relevant articles. In addition, abstracts and presentations from all the major conference proceedings, including the American Society of Clinical Oncology (ASCO), the World Conference on Lung Cancer (WCLC), the European Society for Medical Oncology (ESMO), and the American Association for Cancer Research (AACR), were reviewed. Finally, we reviewed a number of gray literature websites to minimize publication bias. The protocol was registered with the International Prospective Register of Systematic Reviews PROSPERO registration no. CRD42022306449 (further information is listed in **Supplementary Table S1**).

### Study selection

The inclusion criteria were: 1) RCTs or observational trials investigating ICI treatment in advanced lung cancer patients; 2) Available data on hazard ratios (HRs) for overall survival (OS) or progression-free survival (PFS) based on liver status (with or without liver metastases). Exclusion criteria were as follows: 1) Studies without OS and PFS outcomes data according to liver status; 2) Studies that explored only patients with liver metastases or those without liver metastases; 3) Use of an ICI drug in both arms. The most recent and/or most complete trial data were included if duplicate clinical trials were identified. Two authors (Wengang Zhang and Yuqing Zhang) compared the final results, and disagreements about specific studies were discussed and resolved by consensus with all investigators.

### Data extraction

From each study, we extracted the first author and year of publication, region, study design, phase, lung pathology, number of patients, intervention group, control group, treatment lines, and HRs for PFS or OS in patients with/without liver metastases. In addition, for observational trials, to ensure the accuracy of the survival results, we extracted only multivariate analysis survival data.

### Quality assessment and bias assessment

We assessed the quality of randomized trials using the Cochrane Collaboration tool for assessing risk of bias (17), and observational studies using the Newcastle–Ottawa Scale (NOS) (18). Potential publication bias was judged using the Egger test.

### Statistical analyses

Due to the limited number of studies conforming to the inclusion criteria, this meta-analysis was performed by combining the data from studies with different immunotherapy regimens. This approach has been used in previous studies (19). To avoid the risk of ecological bias for RCTs, an interaction trial-specific HR ratio (ratio of the HR in patients with liver metastases to the HR in patients without liver metastases) was calculated for each RCT, and the trial-specific HR ratios were then pooled using the deft approach (20), the feasibility of which has been confirmed by previous studies (21, 22). In order to make the analysis more accurate, this meta-analysis was performed separately for patients with NSCLC and small cell lung cancer (SCLC). To validate the prognostic significance of liver metastasis in advanced lung cancer patients receiving ICI treatment, the collected observational trials were analyzed. We combined the adjusted HRs for OS of patients with liver metastases versus patients without liver metastases.

We assessed the heterogeneity of effects among studies using the χ2- based Q test, which was quantified using the I2 test. For the Q test, P <0.10 was indicated to represent statistically significant heterogeneity, and the I2 statistic represented the amount of total variation that could be attributed to heterogeneity (23). Sensitivity analysis using the “one study removed” approach was performed to determine the sensitivity of the meta‐analysis for each result, as well as inter-study variability. For included RCTs, we conducted subgroup analyses according to the line of therapy. For observational trials, subgroup analyses were performed according to the countries and line of therapy to explore variations in the effect of liver metastases on ICI efficacy.

All statistical analyses were performed using R (version 4.1.2). P-values<0.05 were considered statistically significant.

## Results

### Study selection

**Figure 1:**
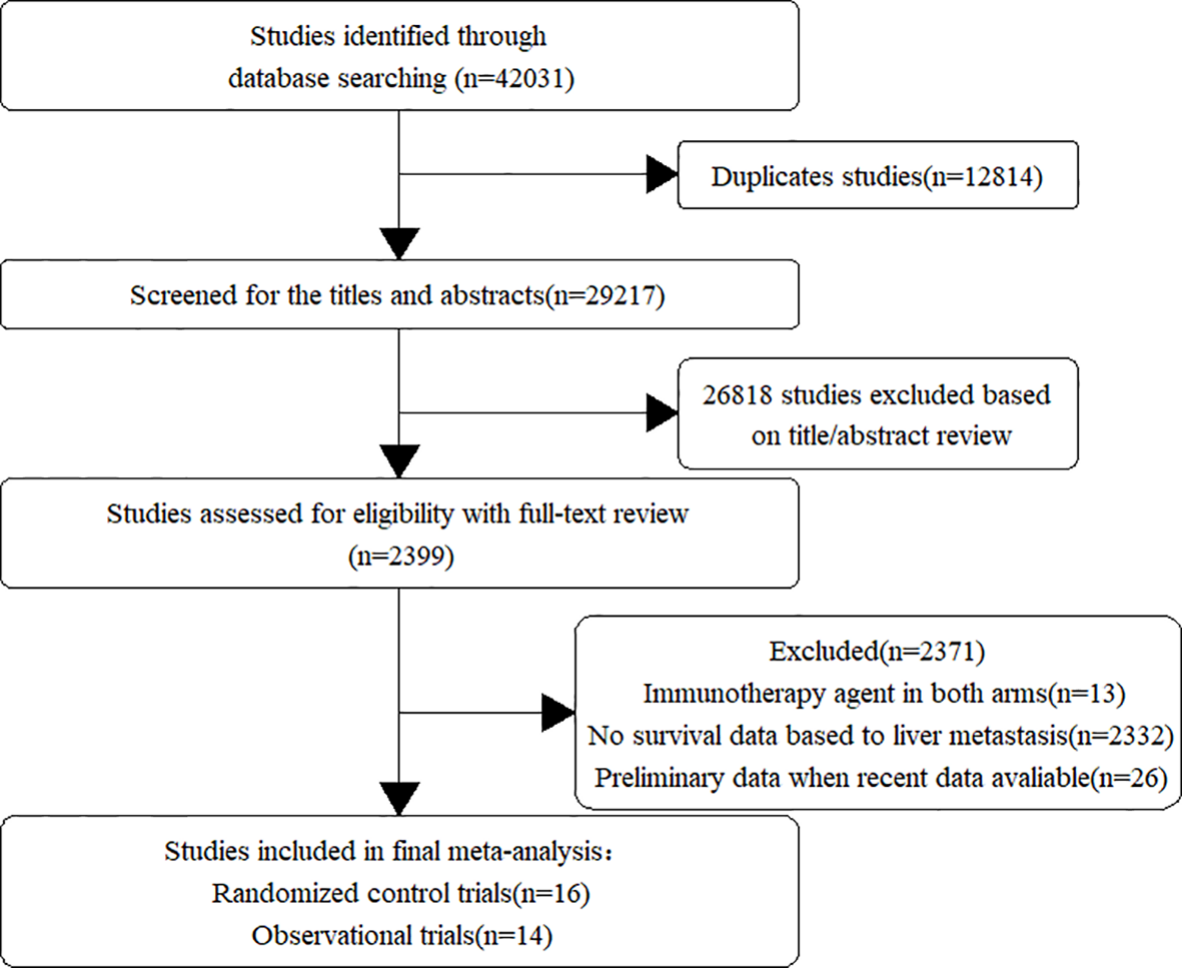
Flow chart of study selection and design.

Our database and manual searches retrieved a total of 42,031 publications, of which 12,814 studies were excluded because of duplication. After full-text screening, we selected 2,399 potentially relevant articles. Finally, we identified 30 informative studies, including 16 RCTs (9, 14, 24–37) and 14 observational trials (12, 13, 38–49) which fulfilled the inclusion criteria (**Table 1**). A flowchart (**Figure 1**) demonstrated the study selection process.

**Table 1:** Summary of studies included in the present meta-analysis.

Author/Year	Region	Study design/Phase	Lung pathology	Therapy line	Intervention (No.)	Control treatment (No.)	LM status (NO.)	
							Yes	No
POPLAR	Wordwide	RCT II	NSCLC	>1	Atezolizumab (n=144)	Docetaxel (n=143)	66	221
OAK	Wordwide	RCT III	NSCLC	>1	Atezolizumab (n=452)	Docetaxel (n=452)	177	673
IMpower131	Wordwide	RCT III	NSCLC	1	Atezolizumab+CT (n=333)	CT (n=340)	139	544
Checkmate227	Wordwide	RCT III	NSCLC	1	Nivolumab+ipilmumab (n=583)	CT (n=583)	252	914
IMpower130	Wordwide	RCT III	NSCLC	1	Atezolizumab+CT (n=451)	CT (n=228)	100	579
IMpower150	Wordwide	RCT III	NSCLC	1	Atezolizumab+CT+bevacizumab (n=400)	Bevacizumab+CT (n=400)	110	690
Checkmate9LA	Wordwide	RCT III	NSCLC	1	Nivolumab+ipilmumab+CT (n=361)	CT (n=358)	154	565
IMpower132	Wordwide	RCT III	NSCLC	1	Atezolizumab+CT (n=292)	CT (n=286)	73	505
NCT03594747	China	RCT III	NSCLC	1	Tislelizumab+CT (n=241)	CT (n=240)	58	423
Keynote189	Wordwide	RCT III	NSCLC	1	Pembrolizumab+CT (n=410)	CT (n=206)	116	500
ORIENT-12	China	RCT III	NSCLC	1	Sintilimab+CT (n=179)	CT (n=178)	14	165
GEMSTONE-302	China	RCT III	NSCLC	1	Sugemalimab+CT (n=320)	Placebo (n=159)	39	18
IMpower133	Wordwide	RCT III	SCLC	1	Atezolizumab+CT (n=201)	CT (n=202)	149	254
Keynote604	Wordwide	RCT III	SCLC	1	Pembrolizumab+CT (n=228)	CT (n=225)	190	263
Checkmate331	Wordwide	RCT III	SCLC	>1	Nivolumab (n=284)	CT (n=285)	205	363
Checkmate451	Wordwide	RCT III	SCLC	>1	Nivolumab+ipilmumab/Nivolumab (n=559)	Placebo (n=275)	325	509
Lobefaro 2021**(** 32 **)**	Italy	Observational trial	NSCLC	un	ICI (n=404)	–	77	327
MengQiao 2021 (33)	China	Observational trial	NSCLC	un	ICI/ICI+CT (n=405)	–	29	203
Diker 2021**(** 34 **)**	Turkey	Observational trial	NSCLC	1	Pembrolizumab+CT/Pembrolizumab (n=406)	–	16	28
Banna 2021**(** 35 **)**	England	Observational trial	NSCLC	1	Pembrolizumab (n=407)	–	23	76
Cortellini 2020**(** 37 **)**	Italy	Observational trial	NSCLC	1	Pembrolizumab (n=410)	–	234	1120
Schouten 2020**(** 38 **)**	Dutch	Observational trial	NSCLC	un	Nivolumab (n=411)	–	43	205
Filippo 2020 (39)	Italy	Observational trial	NSCLC	un	Nivolumab/Pembrolizumab/ Atezolizumab (n=412)	–	29	104
Lim 2020 (40)	Korea	Observational trial	NSCLC	>1	Nivolumab (n=413)	–	32	267
Ahn 2019 (43)	Korea	Observational trial	NSCLC	un	Nivolumab/Pembrolizumab (n=416)	–	24	131
CRINÒ 2019 (31)	Itally	Observational trial	NSCLC	un	Nivolumab (n=417)	–	63	308
Morita 2019 (44)	Japan	Observational trial	NSCLC	>1	Nivolumab (n=418)	–	104	797
Landi 2019 (45)	Italy A	Observational trial	NSCLC	>1	Nivolumab (n=419)	–	327	1261
Landi 2019 (45)	Italy B	Observational trial	NSCLC	>1	Nivolumab (n=420)	–	63	308
Tomoya 2017**(** 49 **)**	Korea	Observational trial	NSCLC	>1	Nivolumab/Pembrolizumab (n=424)	–	10	59
Lee 2021**(** 50 **)**	Korea	Observational trial	SCLC	1	Atezolizumab+CT (n=425)	–	28	68

LM, liver metastases; CT, chemotherapy; NSCLC, non-small-cell lung cancer; SCLC, small-cell lung cancer; Blue indicates RCTs, while gray indicates Observational trials.

### Characteristics of included studies

A total of 16 RCTs were included in this analysis, among which 12 related to NSCLC and four related to SCLC. Four different ICI regimens were used in the included RCTs. Ten of the studies assessed ICI + Chemotherapy, three assessed ICI monotherapy, two assessed dual ICI therapy and only one assessed dual ICI + Chemotherapy. In terms of RCTs of NSCLC, nine trials were conducted worldwide and three trials were conducted in China. Most studies were phase III trials, with the exception of one phase II trial. Ten trials evaluated ICI treatment in the first-line setting, while two trials assessed efficacy in the second- or later-line setting. The number of patients enrolled in each trial ranged between 287 and 1,166. Of the total 7,695 patients included, 1,329 (17%) had liver metastases and 6,366 (83%) had no liver metastases. In terms of RCTs of SCLC, all included studies were international, multicenter phase III trials. Two studies evaluated ICI treatment in the first-line setting, and two assessed the efficacy in the second- or later-line setting. The number of patients enrolled in each trial ranged between 403 and 834. Of the total 2,258 patients included, 869 (38%) had liver metastases and 1,389 (62%) had no liver metastases.

All of the observational studies were single-arm studies. In the included observational studies, twelve studies employed ICI monotherapy and one used ICI + Chemotherapy. The remaining two studies had both ICI monotherapy and combination therapy. Eight studies were conducted in Europe and six were conducted in Asia. Thirteen studies were conducted in patients with NSCLC and only 1 study was conducted in patients with SCLC. The number of patients enrolled in each trial ranged between 44 and 1,588. Of the total 6,364 patients included, 1,102(17%) were patients had liver metastases and 5,262(83%) had no liver metastases. Detailed characteristics of included studies are reported in **Table 1**.

### Relationship between liver metastases and PFS outcomes in patients with NSCLC in RCTs

**Figure 2:**
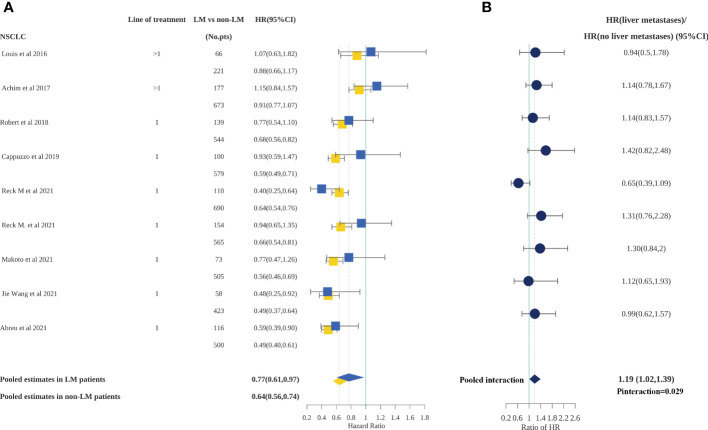
Forest plot of hazard ratios for PFS according to liver metastases in non-small cell lung cancer patients.**(A)**, Hazard Ratios for PFS when comparing immunotherapy to control treatment.**(B)**, The interaction between PFS benefit of immunotherapy and liver metastases.

PFS outcomes for patients with or without liver metastases were available for 12 studies, only one of which included a cohort of patients with SCLC. Therefore, meta-analyses for PFS were conducted in patients with NSCLC only. We calculated the pooled HR for PFS according to the presence or absence of liver metastases**(** **Figure 2** **)**. Patients both with and without liver metastases receiving ICI treatment experienced a significantly lower risk of progression compared with those treated with standard therapies (HR, 0.77; 95%CI, 0.61–0.97 and HR, 0.64; 95%CI, 0.56–0.74, respectively). Furthermore, the difference in ICI efficacy between patients with and without liver metastases was statistically significant (p=0.029) according to an interaction test. The pooled ratio of PFS-HRs in patients with liver metastases versus patients without liver metastases reported in each trial was 1.19 (95% CI, 1.02–1.39); in other words, patients with liver metastases obtained less PFS benefit from ICI treatment than patients without liver metastases. However, the difference became non-significant when sensitivity analysis was performed to separately exclude OAK, IMpower130, and Checkmate9LA, indicating a degree of heterogeneity among the studies**(** **Supplementary Table 3** **)**.

### Relationship between liver metastases and OS outcomes in RCTs

**Figure 3:**
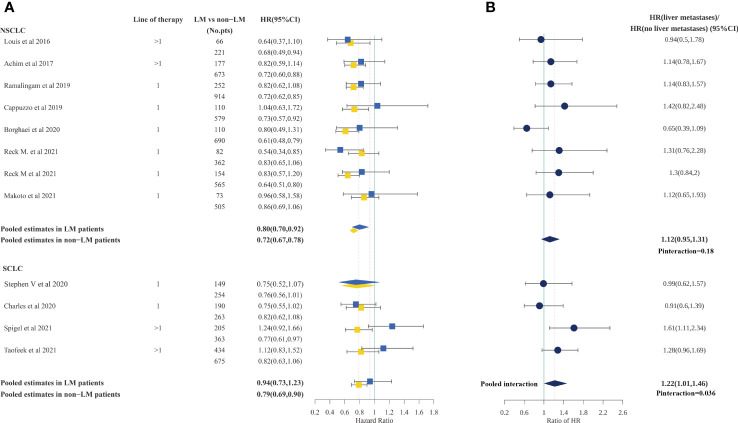
Forest plot of hazard ratios for OS according to liver metastases in non-small cell lung cancer and small cell lung cancer patients.**(A)**, Hazard Ratios for OS when comparing immunotherapy to control treatment.**(B)**, The interaction between OS benefit of immunotherapy and liver metastases.

In patients with NSCLC, we calculated the pooled HR for OS according to the presence or absence of liver metastases based on eight studies (out of 12) that included such information. Consistent with PFS, ICI treatment could reduce the risk of death for patients both with and without liver metastases, compared with standard therapies (HR, 0.78; 95%CI, 0.68–0.90; and 0.72; 95%CI, 0.67–0.78; respectively). However, the difference in ICI efficacy between patients with and without liver metastases was statistically not significant (P=0.24) according to the interaction test. The pooled ratio of OS HRs in patients with liver metastases versus patients without liver metastases reported in each trial was 1.10 (95%CI, 0.94–1.29), suggesting that the OS benefit of immunotherapy was less in NSCLC patients with liver metastases, but not to a statistically significant degree**(** **Figure 3** **)**. Moreover, the sensitivity analysis using a leave-one-out strategy showed no change in result. Notably, the IMpower150 trial demonstrated significant survival improvement in patients with liver metastases and the result did not significantly change after excluding IMpower150**(** **Supplementary Table S3** **)**. Subgroup analyses based on line of therapy also demonstrated no significant differences in the OS outcome**(** **Table 2** **)**.

**Table 2:** Analyses of NSCLC pooled hazard ratios for OS outcomes by subgroup.

Variables	Study, N(%)	Number of patients(N)		Pooled HR (95%CI)		p value for interaction
		LMs	non-LMs	LMs	non-LMs	
Overall	8 (100)	1245	5614	0.78 (0.68,0.90)	0.72 (0.67,0.78)	0.24
Therapy line						
First-line	6 (75)	1002	4720	0.79 (0.67,0.92)	0.72 (0.66,0.79)	0.28
Second- or later-line	2 (25)	243	894	0.77 (0.58,1.02)	0.71 (0.60,0.84)	0.63

NSCLC, non–small cell lung cancer; LMs, liver metastases; OS, overall survival; HR, hazard ratio; CI, confidence interval.

In patients with SCLC, we calculated the pooled HR for OS according to the presence or absence of liver metastases among all four studies which included this information. The analysis demonstrated that patients without liver metastases could benefit from ICI treatment (HR, 0.79; 95%CI, 0.69–0.90), while for patients with liver metastases, the OS benefits were marginal (HR, 0.94; 95%CI, 0.73–1.23). Furthermore, a statistically significant difference in OS was found between patients with liver metastases and patients without liver metastases (P=0.036 for interaction). The pooled ratio of OS HRs in patients with liver metastases versus patients without liver metastases reported in each trial was 1.22 (95%CI, 1.01–1.46)**(** **Figure 3** **)**. However, the sensitivity analysis showed that the difference became non-significant after omitting Checkmate331 (ratio of OS HRs, 1.11; 95%CI, 0.90–1.37) and after omitting checkmate451(ratio of OS HRs, 1.17; 95% CI, 0.92–1.49), indicating a degree of heterogeneity among studies**(** **Supplementary Table S3** **)**. The results of the subgroup analyses for OS outcomes of patients with SCLC are summarized in **Table 3**. Subgroup analyses demonstrated that the difference in OS benefits originated mainly from SCLC patients with second- or later-line immunotherapy (ratios of OS HRs, 1.38; 95%CI, 1.11–1.74; P= 0.0044).

**Table 3:** Analyses of SCLC pooled hazard ratios for OS outcomes by subgroup.

Variables	Study, N(%)	Number of patients(N)		Pooled HR (95%CI)		p value for interaction
		LMs	non-LMs	LMs	non-LMs	
**Overall**	4 (100)	869	1389	**0.94 (0.73,1.23)**	0.79 (0.69,0.90)	**0.036**
**Line of therapy**						
First-line	2 (50)	339	517	0.75 (0.59,0.95)	0.79 (0.65,0.97)	0.7252
Second- or later-line	2 (50)	530	872	**1.09 (0.92,1.30)**	0.79 (0.68,0.91)	**0.0044**

SCLC, small cell lung cancer; LMs, liver metastases; OS, overall survival; HR, hazard ratio; CI, confidence interval. Bold values indicate statisticallysignificant findings with p<0.05.

### Outcomes from real world data

**Figure 4:**
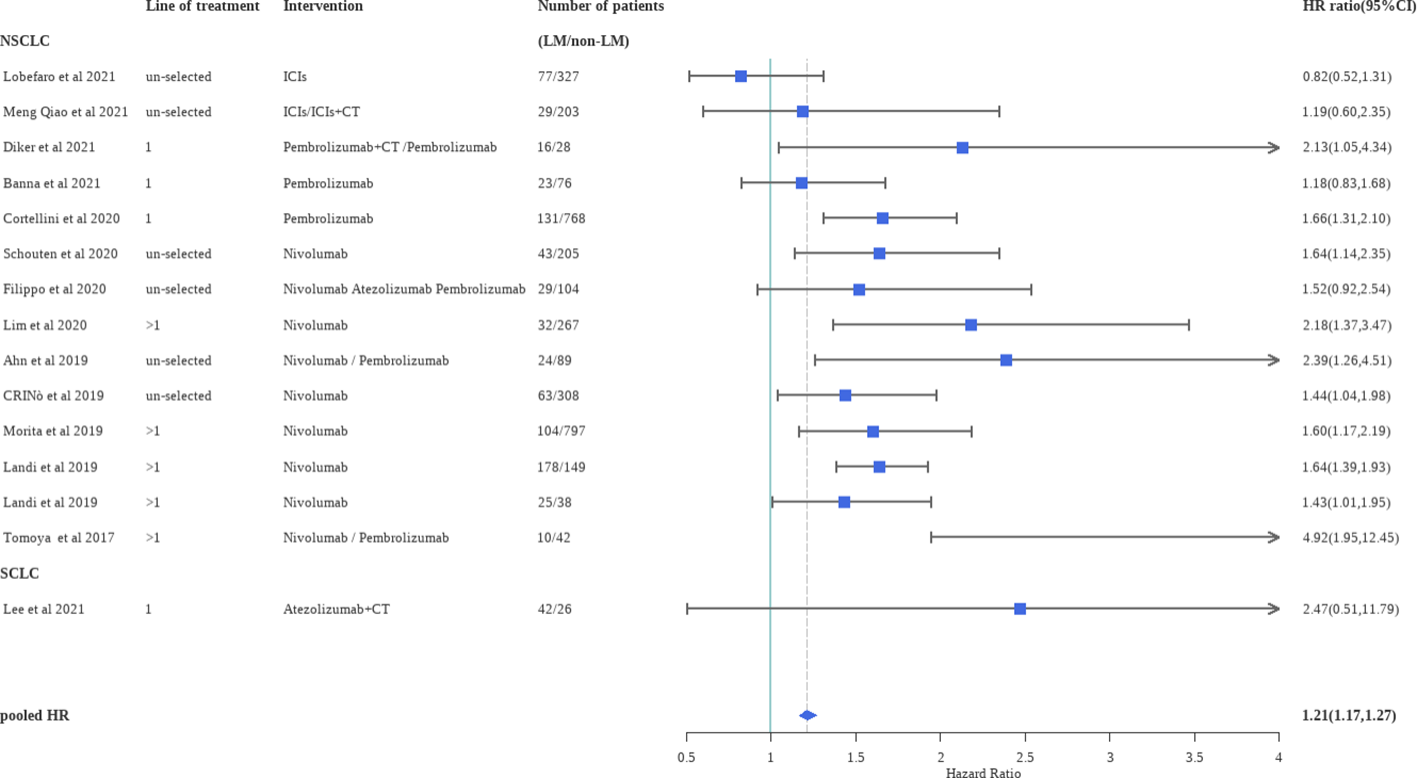
Forest plot of hazard ratios for OS according to liver metastases in clinical practice. ICIs, immune checkpoint inhibitors; CT, chemotherapy.

A total of 14 studies provided multivariate analysis of OS based on liver status. We calculated the pooled HR (patients with liver metastases versus patients without liver metastases) for OS of the included observational trials (HR, 1.21; 95%CI, 1.17–1.27)**(** **Figure 4** **)**. As can be seen in the pooled results, for patients receiving ICI treatment, the presence of liver metastases was an independent prognostic factor associated with worse OS (P<0.0001). Sensitivity analysis, performed by excluding one study at a time, revealed no change in statistical significance. Subgroup analyses of observational studies based on Asian or Western countries and the line of therapy did not show significant differences**(** **Table 4** **)**.

**Table 4:** Analyses of pooled hazard ratios for OS outcomes by subgroup in clinical practice.

Variables	Study, N (%)	Number of patients (LM/non-LM)	Pooled HR (95%CI)	p value of heterogeneity
Overall	14 (100)	826/3427	1.22 (1.17,1.27)	
Line of therapy				
First-line	4 (29)	212/898	1.21 (1.11,1.31)	
Second- or later line	4 (29)	349/1293	1.25 (1.17,1.32)	p=0.33
un-selected	6 (42)	265/1236	1.16 (1.07,1.26)	
Country				
Asian	6 (42)	318/1751	1.28 (1.07,1.53)	p=0.79
Western	8 (58)	508/1676	1.21 (1.16,1.27)	

LMs, liver metastases; OS, overall survival; HR, hazard ratio; CI, confidence interval.

### Publication bias

We performed the Egger’s test for all previous analyses. The results showed that there was no publication bias**(** **Supplementary Table S4** **)**.

## Discussion

The liver is known to have immune regulatory properties, with a unique ability to accept MHC-mismatched allograft (50). The inherent immuno-tolerant characteristics of the liver can lead to an immunosuppressive microenvironment of tumor liver metastasis, which constrains immunotherapy. One of the possible underlying mechanisms is that liver non-parenchymal cells, including hepatic stellate cells (HSCs), macrophages (Kupffer cells), dendritic cells, and liver sinusoidal endothelial cells (LSECs), present antigens to T-cells in a tolerogenic fashion and eventually either tolerize or delete those allospecific T-cells (5). Weiping Zou and his colleagues recently reported that in the liver metastases mouse model, activated antigen-specific Fas+CD8+ T-cells underwent apoptosis after interacting with FasL+CD11b+F4/80+ monocyte-derived macrophages, thereby ultimately reducing immunotherapy efficacy (51). However, a pooled analysis of KEYNOTE-021 cohort G, KEYNOTE-189, and KEYNOTE-407 reported in 2020 (52) indicated that patients with liver metastases could benefit from first-line pembrolizumab plus chemotherapy treatment, although such benefit was relatively lower than in patients without metastases. These conflicting results motivated us to investigate the role of liver metastases in the ICI treatment of lung cancer patients.

This meta-analysis enables us to comprehensively synthesize evidence from both RCTs and observational studies, and to evaluate the interaction between liver metastases and the efficacy of ICI treatment, including ICI monotherapy, ICI + Chemotherapy, dual ICI therapy and dual ICI + Chemotherapy, across different lung cancer patients based on pathology. According to the findings based on included RCTs, ICI treatment in patients with NSCLC could significantly decrease the risk of death and progression for patients with liver metastases. ICI treatment in patients with liver metastases has a comparable effect on OS, but a smaller effect on PFS versus standard therapy, compared with patients without liver metastases. In contrast, ICI treatment exhibits marginal effects on patients with SCLC and liver metastases. ICI treatment in patients with liver metastases has a smaller effect on OS versus standard therapy compared with patients without liver metastases. Further investigation is needed because of the limited data of SCLC. Moreover, liver metastasis is an independent prognostic risk factor and can increase the risk of death in lung cancer patients receiving ICI treatment in clinical practice. This association was not modified by race (Asian vs. Western) or number of treatment lines.

A previous meta-analysis examined the efficacy of PD-1/PD-L1 inhibitors plus chemotherapy in NSCLC patients with or without liver metastases, and reported that the two groups of patients achieved comparable treatment effects on OS, which is consistent with our findings (53). However, the previous meta-analysis only included eight trials, all of which included patients with NSCLC, and did not explore other regimens such as single-agent ICI regimens or dual immunotherapy. Thus, our study provides more evidence on the efficacy of ICI treatment in NSCLC patients with liver metastases. Additionally, we extracted the survival data of ICI treatment in SCLC patients with liver metastases from all available randomized controlled trials so far, which, to the best of our knowledge, has not been performed before. Furthermore, this meta-analysis was limited to datasets from RCTs, which could lack external validity. In this study, we conducted a comprehensive analysis of real-world data and clinically confirmed that liver metastasis is an independent prognostic factor for lung cancer patients receiving ICI treatment. We consider that our study is more reliable because we included a larger number of RCTs and observational studies, resulting in a larger dataset for analysis.

In our subgroup analyses of RCTs, treatment with ICIs significantly improved the OS in any analyses among patients without liver metastases. For patients with liver metastases, the efficacy of ICIs was marginal for patients with NSCLC and SCLC in the second- or later-line treatment setting. In terms of the second- or later-line therapy, we conducted an appropriate test to compare the survival benefit between the group with liver metastases and the group without liver metastases, and the efficacy difference existed only in patients with SCLC. Notably, for the subgroup analyses in the subsequent treatment setting, data were available from only two trials. Thus, the stratified results should be interpreted with some caution due to the limited number of included trials. In addition to that, different regimens may result in different therapeutic effects of immunotherapy. For example, the treatment benefit of the ABCP regimen (i.e., Atezolizumab + Bevacizumab + CT) was more pronounced in NSCLC patients with liver metastases in the IMpower150 trial, although the difference was not statistically significant compared to patients with non-liver metastases (54). However, in this meta-analysis, the subgroup analysis according to treatment regimen was unable to carry out due to limited studies and the variety of immunotherapy regimens included in the studies.

Evidence has been shown that lung cancer patients with liver metastases have a poorer prognosis and higher costs of medical treatment (55). Therefore, development of control programs against such patients appears to be necessary. Until now, there has been insufficient evidence to determine which PD-1 or PD-L1 inhibitors are more appropriate for lung cancer patients with liver metastases. In a recent network meta-analysis of nine RCTs (56), pembrolizumab plus chemotherapy and atezolizumab plus bevacizumab plus chemotherapy provided the most benefit in the treatment of NSCLC patients with liver metastases, in terms of both OS and PFS. Based on these results, large head-to-head trials should be conducted to find the optimal regimen for this type of patient.

In parallel, based on the specific clinical features of lung cancer patients with liver metastases, some potentially promising combination strategies that improve ICI efficacy should be taken into consideration. For example, an anti-angiogenic agent that acts against vascular endothelial growth factor (VEGF) has been approved for the treatment of lung cancer. ECOG4599 (57), the first large phase III clinical study to demonstrate the effectiveness of bevacizumab in combination with chemotherapy in the treatment of lung cancer, showed that patients with liver metastases could achieve better survival after this combination therapy than overall population (OS HR, 0.68 vs 0.79, respectively). Furthermore, with the advent of the immunotherapy era, the final reports of the IMpower150 trial supported the synergistic effect of atezolizumab and bevacizumab combination in non-squamous NSCLC patients with liver metastases (54). A 2021 retrospective study in Australia also demonstrated the feasibility of the IMpower150 regimen in clinical settings (58). Additionally, other therapeutic measures such as immune modulating drugs and radiotherapy also exhibit clinical potential (59–62).

It must be acknowledged that our study has limitations. First, the number of included studies was relatively small, especially for studies including patients with SCLC. The number of studies was insufficient to perform subgroup analyses according to the various ICI regimens, such as ICI monotherapy, ICI + Chemotherapy, dual ICI therapy and dual ICI+ Chemotherapy. Second, this study was performed at the trial level only, and not at the individual level, meaning that variables other than liver metastases could have influenced the effect of ICIs. For example, the subgroup of patients with liver metastases in CheckMate227, there was a significant difference in immunotherapy efficacy between patients with PD-L1 expression≥1% and those with PD-L1 expression<1%(25). Third, the study is also limited by the lack of confirmatory statistical analysis of the safety of ICIs. Adverse effects of ICIs in patients with liver metastases should also be taken into account due to the decreased capacity of the liver to metabolize drugs.

## Conclusion

In conclusion, based on our analyses of RCTs, the presence of liver metastases should not be used to exclude patients with NSCLC from ICI treatment, although these patients may achieve less PFS benefit than those without liver metastases. For patients with SCLC, data from a few studies suggested that the presence of liver metastases may decrease the efficacy of ICI treatment. Moreover, liver metastases should be considered a predictor of poor prognosis in advanced lung cancer patients receiving ICI treatment in clinical practice. More clinical trials are urgently needed to find the best treatment options for advanced lung cancer patients with liver metastases.

## Data availability statement

The original contributions presented in the study are included in the article/**Supplementary Material**. Further inquiries can be directed to the corresponding authors.

## Author contributions

XW and YL conceived and guide the study. HX performed statistical analysis, and drafted the manuscript. WZ, XS and YZ collected the literature, edited figures and revised the manuscript. All authors contributed to the article and approved the submitted version.

## Funding

This work was supported by grants from the National Natural Science Foundation of China (No. 81874044) and the Shandong Provincial Natural Science Foundation (No. ZR2020MH236 and No. ZR2019MH050).

## Conflict of interest

The authors declare that the research was conducted in the absence of any commercial or financial relationships that could be construed as a potential conflict of interest.

## Publisher’s note

All claims expressed in this article are solely those of the authors and do not necessarily represent those of their affiliated organizations, or those of the publisher, the editors and the reviewers. Any product that may be evaluated in this article, or claim that may be made by its manufacturer, is not guaranteed or endorsed by the publisher.

## Abbreviations

NSCLC, non-small cell lung cancer; ICIs, immune checkpoint inhibitors; PD-1/PD-L1, programmed cell death protein 1/ligand 1; CTLs, cytotoxic T lymphocytes; PRISMA, Preferred Reporting Items for Systematic Reviews and Meta-Analyses; RCTs, randomized controlled trials; ASCO, American Society of Clinical Oncology; WCLC, the World Conference on Lung Cancer; ESMO, the European Society for Medical Oncology; AACR, and the American Association for Cancer Research; HRs, hazard ratios; OS, overall survival; PFS, progression-free survival; NOS, Newcastle–Ottawa Scale; SCLC, small cell lung cancer; LM, liver metastases; CT, chemotherapy.
