# Supplementary material for: Liver metastases and the efficacy of immune checkpoint inhibitors in advanced lung cancer: A systematic review and meta-analysis
Source: Front Oncol. 2022 Oct 18;12:978069. doi: 10.3389/fonc.2022.978069 (PMC9623244; doi:10.3389/fonc.2022.978069)
Supplement: Supplementary file 1 [file DataSheet_1.pdf]

Supplementary Online Content

Supplementary table 1. Search Strategies for PubMed, Embase, Cochrane Library and Grey Literature

Supplementary table 2. Risk of Bias of Included (A) Randomized Controlled Trials, (B) Observational Trials

Supplementary table 3. Sensitivity Analysis.

Supplementary table 4. Egger's test for publication bias.

Supplementary table 1. Search Strategies for PubMed, Embase and Cochrane Library

PubMed: 8,230 results

#1 "pembrolizumab" [Supplementary Concept] OR "pembrolizumab" [All Fields] OR "lambrolizumab" [All Fields] OR "Keytruda" [All Fields] OR "MK-3475" [All Fields]

#2 "Nivolumab"[Mesh] OR "Nivolumab"[All Fields] OR "Opdivo"[All Fields] OR "ONO-4538"[All Fields] OR "ONO 4538"[All Fields] OR "MDX-1106"[All Fields] OR "MDX 1106"[All Fields] OR "MDX1106"[All Fields] OR "BMS-936558"[All Fields] OR "BMS 936558"[All Fields] OR "SCH-900475"[All Fields] OR "Nivo"[All Fields]

#3 "atezolizumab"[Supplementary Concept] OR "atezolizumab"[All Fields] OR "anti-PDL1"[All Fields] OR "anti-PD1"[All Fields] OR "Tecentriq"[All Fields] OR "RG7446"[All Fields] OR "RG-7446"[All Fields] OR "MPDL3280A"[All Fields]

#4 "avelumab" [Supplementary Concept] OR "avelumab" [All Fields] OR "MSB0010718C" [All Fields]

#5 "durvalumab" [Supplementary Concept] OR "durvalumab" [All Fields] OR "MEDI-4736" [All Fields] OR "MEDI4736" [All Fields]

#6 "Programmed Cell Death 1 Receptor"[Mesh] OR "PD-1"[All Fields] OR "PD-L1"[All Fields] OR "B7-H1 Antigen"[Mesh] OR "Programmed death ligand 1"[All Fields]

#7 #1 OR #2 OR #3 OR #4 OR #5 OR #6

#8 "Lung Neoplasms"[Mesh] OR "lung cancer*" [Title/Abstract] OR "lung carcinoma*" [Title/Abstract] OR "lung malignan*"[Title/Abstract] OR "lung neoplasm*"[Title/Abstract] OR " lung tumo*"[Title/Abstract] OR "pulmonary cancer*"[Title/Abstract] OR "pulmonary carcinom*"[Title/Abstract] OR "pulmonary malignan*"[Title/Abstract] OR "pulmonary neoplasm*"[Title/Abstract] OR "pulmonary tumo*"[Title/Abstract] OR "carcinoma, non-small-cell lung"[MeSH] OR "nonsmall cell lung cancer*"[Title/Abstract] OR "non small cell lung cancer*"[Title/Abstract] OR "non small cell lung carcinoma*"[Title/Abstract] OR "NSCLC"[Title/Abstract] OR "carcinoma, small cell" [MeSH] OR "oat cell carcinoma*"[Title/Abstract] OR "oat cell lung carcinoma*"[Title/Abstract] OR "oat cell lung cancer*"[Title/Abstract] OR "oat cell cancer*"[Title/Abstract] OR "SCLC" [Title/Abstract] OR "small cell lung cancer*"[Title/Abstract] OR "small cell lung carcinom*"[Title/Abstract]

#9 animals [mh] NOT humans [mh]

#10 #7 AND #8 NOT #9

EMBASE:26,324results

#1 'pembrolizumab'/exp

#2 'pembrolizumab' OR 'lambrolizumab' OR 'keytruda' OR 'mk-3475'

#3 **'**nivolumab'/exp

#4 'nivolumab' OR 'opdivo' OR 'ono-4538' OR 'ono 4538' OR 'mdx-1106' OR 'mdx 1106' OR 'mdx1106' OR 'bms-936558' OR 'bms 936558' OR 'sch-900475' OR 'nivo'

#5 'atezolizumab'/exp

#6 'atezolizumab' OR 'anti-pdl1' OR 'tecentriq' OR 'rg7446' OR 'rg-7446' OR 'mpdl3280a'

#7 'avelumab'/exp

#8 'avelumab' OR 'msb0010718c'

#9 'durvalumab'/exp

#10 'durvalumab' OR 'medi-4736' OR 'medi4736'

#11 'programmed cell death 1 receptor'/exp

#12 'programmed cell death 1 receptor' OR 'pd-1' OR 'pd-l1' OR 'programmed death ligand 1'

#13 'b7-h1 antigen'/exp

#14 #1 OR #2 OR #3 OR #4 OR #5 OR #6 OR #7 OR #8 OR #9 OR #10 OR #11 OR #12 OR #13

#15 'lung tumor'/exp

#16 'lung cancer*':ab,ti OR 'lung carcinoma*':ab,ti OR 'lung malignan*':ab,ti OR 'lung neoplasm*':ab,ti OR 'lung tumo*':ab,ti OR 'pulmonary cancer*':ab,ti OR 'pulmonary carcinom*':ab,ti OR 'pulmonary malignan*':ab,ti OR 'pulmonary neoplasm*':ab,ti OR 'pulmonary tumo*':ab,ti

#17 #15 OR #16

#18 'lung non small cell cancer'/exp

#19 'nonsmall cell lung cancer*':ab,ti OR 'non small cell lung cancer*':ab,ti OR 'nonsmall cell lung carcinoma*':ab,ti OR 'non small cell lung carcinoma*':ab,ti OR 'nsclc':ab,ti

#20 #18 OR #19

#21 'small cell lung cancer'/exp

#22 'oat cell carcinoma*':ti,ab OR 'oat **cell lung carcinoma*'**:ti,ab OR **'oat cell lung cancer*'**:ti,ab OR **'oat cell cancer*'**:ti,ab OR **'sclc'**:ti,ab OR **'small cell lung cancer*'**:ti,ab OR **'small cell lung carcinom*'**:ti,ab

#23 #21 OR #22

#24 #17 OR #20 OR #23
#25 #14 AND #24

Cochrane library: 2,298 results
#1 MeSH descriptor: [Lung Neoplasms] explode all trees
#2 （lung cancer*） :ti,ab,kw OR (lung carcinoma*):ti,ab,kw OR (lung malignan *):ti,ab kw OR (lung neoplasm*):ti,ab kw OR (lung tumo*):ti,ab,kw
#3 （pulmonary cancer*） :ti,ab,kw OR (pulmonary carcinom*):ti,ab,kw OR (pulmonary malignan*):ti,ab kw OR (pulmonary neoplasm*):ti,ab kw OR (l pulmonary tumo*):ti,ab,kw
#4 #1 or #2 or #3
#5 MeSH descriptor: [Carcinoma, Non-Small-Cell Lung] explode all trees
#6 （nonsmall cell lung cancer*） :ti,ab,kw OR (non small cell lung cancer*):ti,ab,kw OR (nonsmall cell lung carcinoma*):ti,ab kw OR (non small cell lung carcinoma*):ti,ab kw OR (NSCLC):ti,ab,kw
#7 #5 or #6
#8 MeSH descriptor: [Carcinoma, Small Cell] explode all trees
#9 （oat cell carcinoma*） :ti,ab,kw OR (oat cell lung carcinoma*):ti,ab,kw OR (oat cell lung cancer*):ti,ab kw OR (oat cell cancer*):ti,ab kw OR (SCLC):ti,ab,kw
#10 (small cell lung cancer*） :ti,ab,kw OR (small cell lung carcinom*):ti,ab,kw
#11 #8 or #9 or #10
#12 #4 or #7or #11
#13 MeSH descriptor: [Nivolumab] explode all trees
#14 （pembrolizumab OR lambrolizumab OR Keytruda OR MK-3475） :ti,ab,kw OR (PD-1 or PD-L1 or programmed cell death 1 receptor or programmed death ligand 1):ti,ab,kw
#15 MeSH descriptor: [B7-H1 Antigen] explode all trees
#16 MeSH descriptor: [Programmed Cell Death 1 Receptor] explode all trees
#17 （Nivolumab or Opdivo OR ONO-4538 OR ONO 4538 OR MDX-1106 OR MDX 1106 OR MDX1106 OR BMS-936558 OR BMS 936558 OR SCH-900475 OR Nivo） :ti,ab,kw OR (atezolizumab OR Tecentriq OR RG7446 OR RG-7446 OR MPDL3280A):ti,ab,kw OR (avelumab OR MSB0010718C):ti,ab kw OR (durvalumab OR

MEDI-4736 OR MEDI4736):ti,ab kw
#18 #13 or #14 or #15 or #16 or #17
#19 #12 and #18

Grey Literature

Grey Literature Report (GreyLit Net-work)

http://www.greylit.org/ **search terms:** lung cancer AND immunotherapy

Grey Horizon

http://grey − horizon.blogspot.nl/ **search terms:** lung cancer

Grey Matters: A Practical Search Tool for Evidence-Based Medicine

https://www.cadth.ca/grey-matters **search terms:** lung cancer AND immunotherapy

Supplementary table 2.

(A) Risk of bias of included randomized controlled trials

**Study**	**Randomisation sequence generation (selection bias)**	**Allocation concealment**	**Blinding**	**withdrawal/discontinuation**	**Jadad Scale**
Louis et al	2	2	0	1	5
Achim et al	2	2	0	1	5
Robert et al	1	1	0	1	3
Ramalingam et al	1	1	0	0	2
Cappuzzo et al	2	1	0	1	4
Brahmer et al	1	1	0	1	3
Borghaei et al	2	2	2	1	7
Reck et al	2	1	0	1	4
Nakagawa et al	1	1	2	1	4
Reck et al	2	2	0	1	5
Makoto et al	2	2	0	1	5
Jie et al	2	1	0	1	4
Abreu et al	2	2	2	1	7
Stephen et al	1	1	2	1	5
Charles et al	2	1	2	1	6
Spigel et al	2	1	0	1	4
Taofeek et al	1	1	2	1	4

(B) Risk of bias of included observational trials

**Supplementary table 3. Risk of bias of in cluded Study**	**Representativeness of the exposed cohort**	**Selection of the non exposed cohort**	**Ascertainment of exposure**	**Demonstration that outcome of interest was not present at start of study**	**Comparability of cohorts on the basis of the design or analysis**	**Assessment of outcome**	**Was follow-up long enough for outcomes to occur**	**Adequacy of follow up of cohorts**	**Quality scores**
Lobefaro et al	1	1	1	1	0	1	1	1	7
Qiao et al	1	1	1	1	0	1	0	1	6
Diker et al	1	1	1	1	1	1	0	1	7
Banna et al	1	1	1	1	0	1	1	1	7
Cortellini et al	1	1	1	0	0	1	1	1	6
Schouten et al	1	1	1	1	1	1	1	1	8
Dall'Olio et al	1	1	1	0	1	1	1	1	7
Sun et al	1	1	1	0	0	1	1	1	6
Ahn et al	1	1	1	1	0	1	1	1	7
CRINÒ et al	1	1	1	0	0	1	0	1	5
Morita et al	1	1	1	1	0	1	1	1	7
Landi et al	1	1	1	0	1	1	0	1	6
Landi et al	1	1	1	0	0	1	0	1	5
Fukui et al	1	1	1	1	0	1	1	1	7

Supplementary table 3. Results of sensitivity analyses

Tumor pathology	Removed studies	Outcome	The pooled ratio of HR
NSCLC	OAK	PFS	1.17(0.99,1.40)
NSCLC	IMpower130	PFS	1.15(0.98,1.36)
NSCLC	Checkmate9LA	PFS	1.16(0.98,1.37)
NSCLC	IMpower150	OS	1.16(0.98,1.38)
SCLC	Checkmate331	OS	1.11(0.90,1.37)
SCLC	Checkmate451	OS	1.17(0.92,1.49)

Supplementary table 4. Egger's test for publication bias.

Outcome	P
Ratio of PFS-HRs for NSCLC	p = 0.359
Ratio of OS-HRs for NSCLC	p = 0.548
Ratio of OS-HRs for SCLC	p = 0.750
OS-HR from real world data	p = 0.544
